# Comparative Analysis and Identification of miRNAs and Their Target Genes Responsive to Salt Stress in Diploid and Tetraploid *Paulownia fortunei* Seedlings

**DOI:** 10.1371/journal.pone.0149617

**Published:** 2016-02-19

**Authors:** Guoqiang Fan, Xiaoyu Li, Minjie Deng, Zhenli Zhao, Lu Yang

**Affiliations:** Institute of Paulownia, Henan Agricultural University, 450002 Zhengzhou, Henan, P.R. China; East Carolina University, UNITED STATES

## Abstract

Salt stress is a global environmental problem that affects plant growth and development. *Paulownia fortunei* is an adaptable and fast-growing deciduous tree native to China that is environmentally and economically important. MicroRNAs (miRNAs) play important regulatory roles in growth, development, and stress responses in plants. MiRNAs that respond to biotic stresses have been identified; however, how miRNAs in *P*. *fortunei* respond to salt stress has not yet been reported. To identify salt-stress-responsive miRNAs and predict their target genes, four small RNA and four degradome libraries were constructed from NaCl-treated and NaCl-free leaves of *P*. *fortunei* seedlings. The results indicated that salt stress had different physiological effects on diploid and tetraploid *P*. *fortunei*. We detected 53 conserved miRNAs belonging to 17 miRNA families and 134 novel miRNAs in *P*. *fortunei*. Comparing their expression levels in diploid and tetraploid *P*. *fortunei*, we found 10 conserved and 10 novel miRNAs that were significantly differentially expressed under salt treatment, among them eight were identified as miRNAs probably associated with higher salt tolerance in tetraploid *P*. *fortunei* than in diploid *P*. *fortunei*. Gene Ontology and Kyoto Encyclopedia of Genes and Genomes pathway analyses were performed to predict the functions of the target genes of the conserved and novel miRNAs. The expressions of 10 differentially expressed miRNAs were validated by quantitative real-time polymerase chain reaction (qRT-PCR). This is the first report on *P*. *fortunei* miRNAs and their target genes under salt stress. The results provided information at the physiological and molecular levels for further research into the response mechanisms of *P*. *fortunei* to salt stress.

## Introduction

MicroRNAs (miRNAs) are endogenous non-coding RNAs that are from 18 to 25 nucleotides (nt) in length. They are found mostly in eukaryons[1–4]. MiRNAs play important roles in regulating gene expression at the transcriptional and posttranscriptional levels. They bind to target mRNAs to form RNA-induced silencing complexes (RISC) that cleave or inhibit their translation[5]. MiRNAs are known to be involved in growth, development, hormone regulation, stress responses, and signal transduction [1, 6].

*Paulownia fortunei*, a member of the family Scrophulariaceae, is one of the most adaptable and fast-growing deciduous trees native to China. Biotic and abiotic stresses are the main factors that affect *Paulownia* growth, biological yield, and timber quality. Salt stress is one of the gravest abiotic stresses that affect this tree. Tetraploid *paulownia* trees, which possess two sets of the same chromosomes, have better timber quality and improved stress resistance than diploid *Paulownia*[7, 8]. In 2007, autotetraploid *P*. *fortunei* seedlings were firstly induced from diploid plants using colchicines to increase genetic diversity [9]. Since then, a number of studies have revealed the morphology and molecular biology of diploid and tetraploid *Paulownia* plants. Physiological biochemical indices such as leaf relative conductivity and superoxide dismutase (SOD) activity have been reported for *Paulownia*[10–12], and information about miRNAs and their target genes have been analyzed in *P*. *fortunei*, *P*. *tomentosa*×*P*. *fortunei*, *P*. *tomentosa*, and *P*. *australis* using high-throughput sequencing approaches[13–17]. In addition, some studies have reported results for various *Paulownia* species under abiotic stresses [18–20].

In this study, we used high-throughput sequencing technology to identify and analyze the expression levels of conserved and novel miRNAs in diploid and tetraploid *P*. *fortunei* under salt stress. We also used degradome analysis to investigate the potential roles of miRNA target genes. The aim of the study was to combine small RNA(sRNA) sequencing with degradome sequencing to identify potential *Paulownia*-specific miRNAs and their targets in four *P*. *fortunei* libraries.

## Materials and Methods

### Plant materials and treatments

Diploid and tetraploid *P*. *fortunei* seedlings used in this study were obtained from the Institute of *Paulownia*, Henan Agricultural University, China. The seedlings were culture-grown on 1/2 Murashige-Skoog(MS) medium with 20g·L^−1^ sucrose under a 16/8 h(light/dark) photoperiod at 25°C. After 30 days (i.e., on 20 April 2012), the test-tube plantlets were transplanted outside, and after another 30 days, the surviving seedlings were transplanted into 30-cmpotswith common garden soil nutrition (each pot contained the same soil weight). The seedlings with the greatest growth consistency were chosen in July2012 for use in this study.

Soil salinity was expressed as the percentage of NaCl weight/dry soil weight. The concentration gradient of NaCl was 0.2%, 0.4%, and 0.6% in treated plantlets; untreated plantlets (0% NaCl) were used as controls. For the salt treatment, NaCl was weighed for each pot and divided into three equal parts. Then, one part was dissolved in water and poured into the pot (a salver was placed under the pot and any water that percolated out from soil was poured back into the pot). This step was repeated three times. The plants were treated in this way every 3days.At the same time, the pots with untreated plantlets were irrigated with the same amount of water. After adding NaCl, the pots were watered every 2 days to keep the soil moisture content at 75% of field capacity. After 20 days, the second pair of leaves from the apiculus of the seedlings were collected and stored at −80°C for further study.

### Determination of physiological indices

Physiological characteristics of diploid and tetraploid *P*. *fortunei* seedlings that were treated with 0%, 0.2%, 0.4%, and 0.6% saltmass fractions were measured between 8.30 am and 11.00 am on single, fully expanded leaves (i.e., the second pair of leaves from the apiculus of the seedlings) immediately after excision. The measurements were taken on three replicates of the leaves from the same plants. The leaf relative water content was measured according to Liu et al.[21], and the leaf chlorophyll content, leaf relative conductivity, malondialdehyde (MDA) content, SOD activity of the leaves, soluble protein content, soluble sugar content, and proline content were determined using the methods reported by Lu et al.[22].

### Small RNA library construction and sequencing

Total RNA was extracted from PF2U (diploid *P*. *fortunei* with 0% NaCl), PF2S (diploid *P*. *fortunei* with 0.4% NaCl), PF4U (tetraploid *P*. *fortunei* with 0% NaCl), and PF4S (tetraploid *P*. *fortunei* with 0.4% NaCl) leaves using Trizol reagent (Invitrogen, Carlsbad, CA) according to the manufacturer’s instructions. Fours RNA libraries were constructed on the GAIIx platform (Illumina, San Diego, CA, USA). The cDNA libraries were purified by polyacrylamide gel electrophoresis (PAGE) and fragments from 140nt to 160nt in length were selected and sequenced on the GAIIx platform (Illumina)following the manufacturer’s standard cBot and sequencing protocols. Briefly, 4μg of total RNA was ligated to adapters sequentially, reverse transcribed to create single-strand cDNA, and amplified by PCR (12 cycles).

### Identification of miRNAs

The raw reads that were obtained by Illumina sequencing were filtered to remove reads that contained adapter sequences, contaminated reads, and low-quality reads. The length distribution of the unique clean reads was analyzed and then mapped onto the Paulownia UniGene sequences (http://trace.ncbi.nlm.nih.gov/Traces/sra/) [23] using miRDeep2 [24] to acquire perfectly matched reads. The tRNA, rRNA, snoRNA, and ncRNA were detected by Blastall (http://www.ncbi.nlm.nih.gov/staff/tao/URLAPI/blastall/) against the Non-coding RNA Database (Release 10; http://rfam.sanger.ac.uk/) and removed from the *P*. *fortunei* library datasets. The remaining sRNA reads were searched using Blastall (http://www.ncbi.nlm.nih.gov/staff/tao/URLAPI/blastall/) (allowing two mismatches) against the plant mature miRNA sequences in miRBaseRelease 21.0 to identify conserved miRNAs. To identify potential novel miRNAs, MIREAP (http://sourceforge.net/projects/mireap/) and RNA fold (http://rna.tbi.univie.ac.at/cgi-bin/RNAfold.cgi) were used to fold flanking sequences and predict secondary structures of the unmatched sRNAs. The sRNAs were considered to be candidate novel miRNAs if they formed perfect stem-loop structures and followed the other criteria described by Meyers et al.[25].

### Differential expression analysis of miRNAs in the four *P*. *fortunei* libraries

The sequence reads from the PF2U, PF2S, PF4U, and PF4S libraries were used to analyze the differential expression of the miRNAs. The miRNAs identified in the four libraries were normalized to one million [normalized expression = (number of miRNA reads/total number of clean reads)*1,000,000]; normalized miRNA expression values<1were removed.

The fold change was obtained according to the calculations as follows:

Fold change = log_2_ (one normalized miRNA reads / another normalized miRNA reads)

The *P*-value was obtained according to the calculations as follows:

$$P\left(x|y\right)=(\frac{{}_{N_{2}}}{N_{1}})\frac{\left(x+y\right)!}{x!y!{\left(1+\frac{N_{2}}{N_{1}}\right)}^{\left(x+y+1\right)}}$$

$$C\left(y\leq y_{\text{min}}|x\right)=\sum_{y=0}^{y\leq y_{\text{min}}}\text{p}(y|x)$$

$$D\left(y\geq y_{\text{max}}|x\right)=\sum_{y\geq y_{\text{max}}}^{\infty }\text{p}(y|x)$$

Where N_1_and N_2_ represent the total number of clean reads in PF2S and PF2U (or PF4U and PF2U, or PF4S and PF4U, or PF4S and PF2S), respectively; x and y represent the number of miRNAs surveyed in PF2S and PF2U (or PF4U and PF2U, or PF4S and PF4U, or PF4S and PF2S), respectively; C and D can be regarded as the probability discrete distribution of the *P*-value inspection.

### Identification of miRNA target genes by degradome sequencing

To predict genes that may be regulated by the miRNA in the PF2U, PF2S, PF4U, and PF4Splants, four degradome libraries suitable for miRNA target identification were constructed according to the protocol described previously [26, 27]. Briefly, poly (A) RNA was isolated and ligated to a 5′ RNA adapter containing a *Mme*I (NEB, Ipswich, MA, USA) recognition site. After reverse transcription using oligo(dT) and PCR enrichment, the PCR products were purified and digested with *Mme*I. Next, a double-strand DNA adapter was ligated to the digested products using T4 DNA ligase (NEB, Ipswich, MA). The products were amplified using 20 PCR cycles and purified to obtain the final cDNA library, which was sequenced on an Illumina HiSeq™ 2000 system.

After initial processing, the unique sequence signatures were mapped to the *P*. *fortunei* transcriptome sequences (http://soap.genomics.org.cn/) using the SOAP (http://soap.genomics.org.cn/) software to define the coverage rate. Sequences that perfectly matched a transcriptome sequence were retained and extended by adding approximately 15 nt of the upstream sequence to the 31-nt-long signatures. All resulting sequences were reverse complemented and aligned to the corresponding miRNA sequence. Alignments with scores ≤4 and with the 5′ end of the degradome sequence mapped to the tenth and eleventh nucleotides of the complementarity miRNA sequence were considered as potential targets. Additionally, t-plots were built according to the distribution of signatures (and abundances) along the transcriptome sequences. To better understand the functions of these targets, the best homologs were applied to gain the gene ontology (GO) annotations (http://www.geneontology.org/). The pathways were performed based on Blastall hits against the Kyoto Encyclopedia of Genes and Genomes (KEGG) databases (http://www.genome.jp/kegg/) by an *E*-value threshold of <10^−5^.

### Quantitative real-time PCR analysis of miRNA expression

Some of the *P*. *fortunei* miRNAs and their target genes were experimentally validated using quantitative real-time PCR (qRT-PCR). The RNAs from three biological replications were used for qRT-PCR, and total RNA was isolated using a Plant RNA Extraction Kit (Aidlab Biotechnologies Co., Ltd., Beijing, China). For qRT-PCR, stem-loop primers were designed as described previously[28]. The forward primers were designed based on the mature miRNA sequences and the reverse primers were the universal reverse primers, with U6 as the endogenous reference. For the candidate target genes, the primers were designed using Beacon Designer (version 7.7) (Premier Biosoft International, Ltd., Palo Alto, CA, USA) with 18S rRNA as the endogenous reference. All the PCR reactions were run in triplicate for each sample. The primers sequences are listed in S1 and S2 Tables. A SuperScriptIIIPlatinum SYBR Green One-Step qRT-PCR kit (Invitrogen, Carlsbad, CA, USA) and a CFX96 real time PCR system (Bio-Rad) were used to detect and compare the expression levels. For each reaction, 500ng of total RNA was mixed with 10mL of SuperScriptIIIPlatinum SYBR Green PCR master mix and 4pmol each of the reverse transcription forward and reverse primers in a final volume of 20mL. For the PCR amplifications, the conditions were as follows: 40 cycles at 95°C for 15s and 55°C for 30s. The 2^−ΔΔCT^relative quantization method was used to analyze relative changes in gene expression.

## Results

### Physiological responses to salt stress

**Fig 1:**
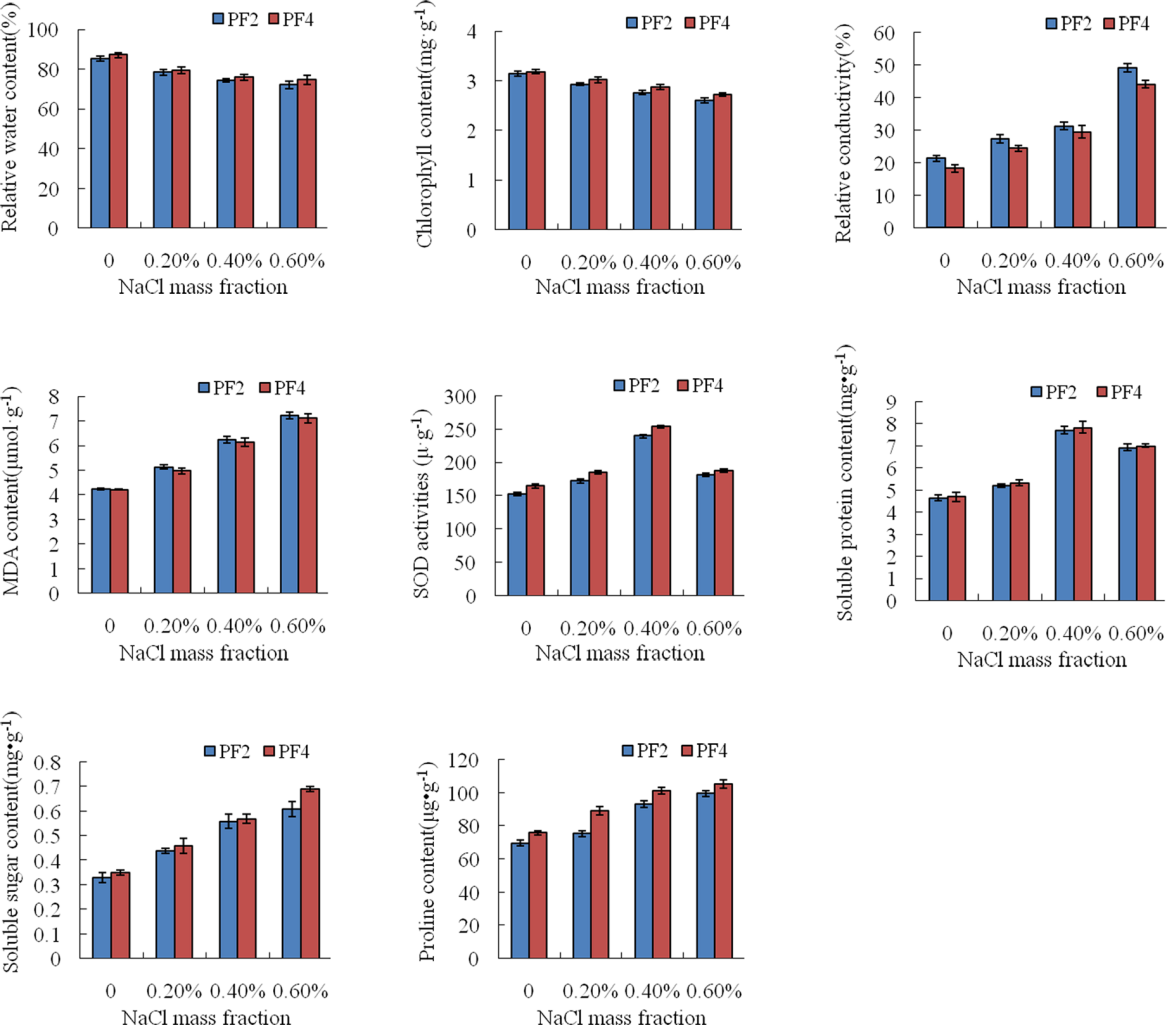
Determination of physiological indices. PF2:diploid *P*. *fortunei*; PF4: tetraploid *P*. *fortunei*. Standard error of the mean for three technical replicates is represented by the error bars.

The relative water contents of the leaves of diploid and tetraploid *P*. *fortunei* declined progressively from 85.23% to 74.65% and from 87.23% to 74.65%, respectively, in the 0%, 0.2%, 0.4%, and 0.6% salt-treated plants (Fig 1). At the same salinity, the relative water content in tetraploid was higher than that in diploid. The chlorophyll contents of the leaves followed the same trend as the relative water contents; that is, with increasing salt concentrations, the chlorophyll content of the leaves declined progressively from 3.15mg·g^−1^ to 2.61mg·g^−1^ and from 3.19mg·g^−1^ to 2.73mg·g^−1^in diploid and tetraploid *P*. *fortunei*, respectively. At the same salinity, tetraploid contained higher chlorophyll content than diploid (Fig 1). The contents of relative conductivity and MDA in leaves generally increased with increasing salt concentrations. Relative conductivity increased progressively from 21.29% to 49.23% and from 18.23% to 44.12%in diploid and tetraploid, respectively; and MDA content of leaves increased progressively from 4.23μmol·g^−1^ to 7.23μmol·g^−1^andfrom 4.21μmol·g^−1^ to 7.11μmol·g^−1^in diploid and tetraploid, respectively (Fig 1). The soluble sugar content in leaves increased progressively from 0.33mg·g^−1^ to 0.61mg·g^−1^and from 0.35mg·g^−1^ to 0.69mg·g^−1^in diploid and tetraploid, respectively. The proline content of leaves increased progressively from 69.8μg·g^−1^ to 99.7μg·g^−1^and from 75.9μg·g^−1^ to 105.3μg·g^−1^in diploid and tetraploid, respectively (Fig 1). At the same salinity, the relative conductivity and MDA content of leaves in diploid plants were always higher than in tetraploid plants, while the contents of soluble sugar and proline in leaves were always higher in tetraploid compared with diploid.

In addition, when the mass fraction of salt ranged from 0% to 0.4%, SOD activity in the leaves increased progressively from 152.5U·g^−1^to 239.5U·g^−1^and from 164.4U·g^−1^to 253.4U·g^−1^in diploid and tetraploid, respectively. The soluble protein content increased progressively from 4.66mg·g^−1^to 7.73mg·g^−1^ and from 4.71mg·g^−1^to 7.84mg·g^−1^in diploid and tetraploid, respectively. When the mass fraction of salt was 0.6%, SOD activity began to decrease to 181.2U·g^−1^and 187.4U·g^−1^in diploid and tetraploid, respectively. Soluble protein content also began to decrease to 6.93mg·g^−1^ and 7.01mg·g^−1^in diploid and tetraploid, respectively (Fig 1). At the same salinity, the contents of SOD activity and soluble protein of the leaves were higher in tetraploid than in diploid.

### Statistical analysis of sRNAs

**Fig 2:**
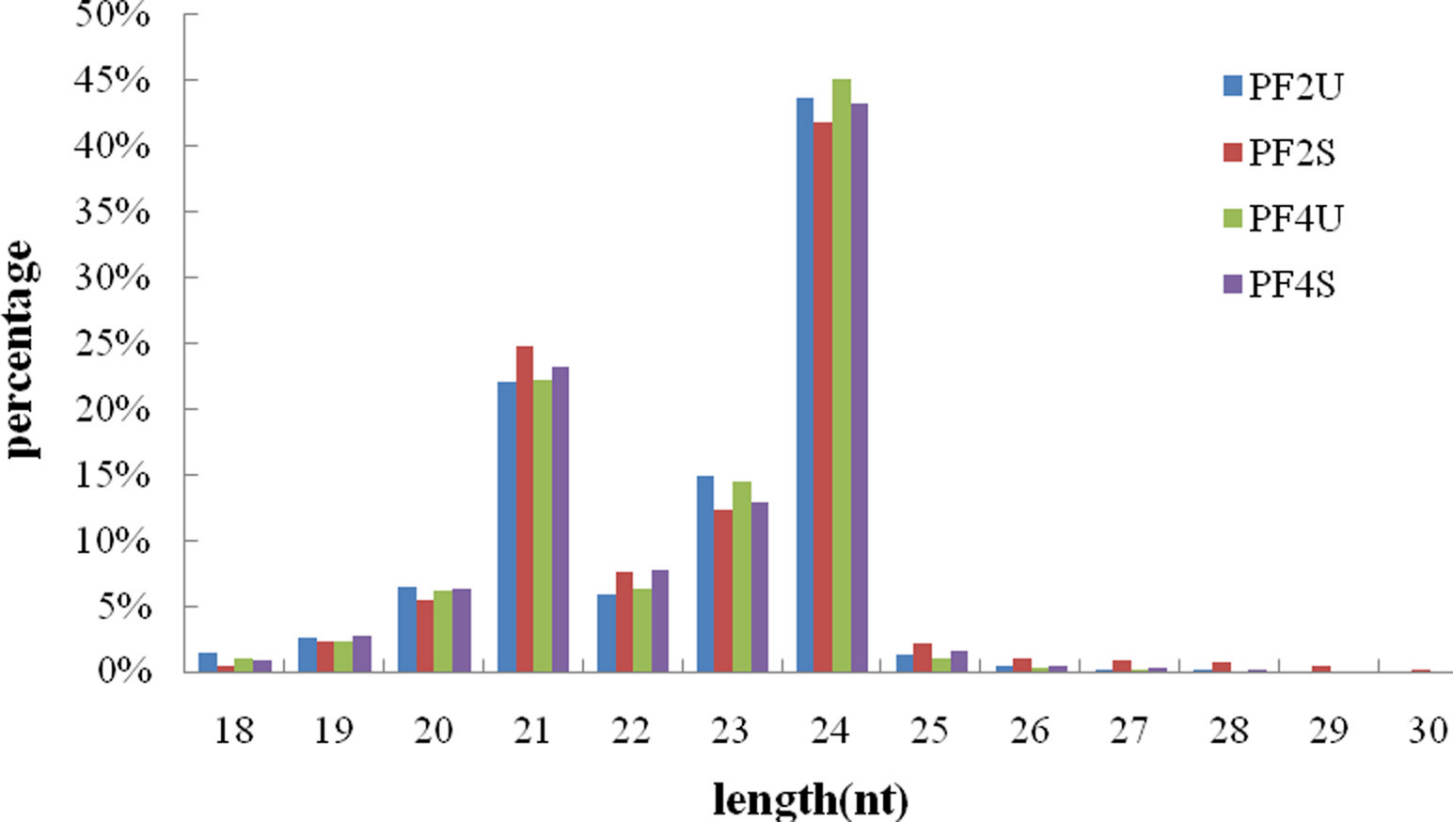
Length (nt) distribution of sRNAs. PF2U: diploid *P*. *fortunei* with0% NaCl; PF2S: diploid *P*. *fortunei* with 0.4% NaCl; PF4U: tetraploid *P*. *fortunei* with0% NaCl; PF4S: tetraploid *P*. *fortunei* with 0.4% NaCl.

A total of 17,224,782 (PF2U), 17,815,289 (PF2S), 15,230,320 (PF4U), and 18,891,961 (PF4S) reads were generated from the four sRNA libraries by Illumina sequencing. After removing low-quality tags, adaptors, contaminants, sequences shorter than 18nt, and sequences with poly-A tails, 16,961,959(PF2U), 17,685,354(PF2S), 15,021,455(PF4U), and 18,729,181(PF4S) clean reads remained for further analysis (Table 1). Among all four libraries, clean reads of 21–24 nt were the most abundant (Fig 2). The 24-nt-long reads made up an average of approximately 44% of all the reads in the four libraries, followed by 21-nt-long reads (approximately 23%), 23-nt-long reads (approximately 14%), and 22-nt-long reads (approximately7%). As expected, the abundances of the23-and 24-nt-long sRNAs in the salt-treated libraries were less than in the untreated libraries. However, the abundances of the21- and 22-nt-long sRNAs in the salt-treated libraries were more than in the untreated libraries, indicating that the numbers of 21- and 22-nt-longsRNAs increased under salt stress. The length distributions of the sRNAs in diploid and tetraploid *P*. *fortunei* libraries were similar, which indicated that the double chromosome played only a small role in the length distribution of the sRNAs. Additionally, sRNA sequences that mapped to the *P*. *fortunei* genome were classified as either conserved miRNAs (if the matched sequences in miRBase) or non-annotated sRNAs, which were used to identify candidate novel miRNAs.

**Table 1:** Annotation of sRNAs sequences.

category		total	miRNA	rRNA	snRNA	snoRNA	tRNA	unannote
PF2U	Unique sRNAs	5392667	18131	44693	1637	694	9468	5318044
	Percent%	100%	0.34%	0.83%	0.03%	0.01%	0.18%	98.62%
	Total sRNAs	16961959	2887692	579878	3064	1526	567569	12922230
	Percent%	100%	17.02%	3.42%	0.02%	0.01%	3.35%	76.18%
PF2S	Unique sRNAs	5605552	18323	68536	2931	911	15976	5498875
	Percent%	100%	0.33%	1.22%	0.05%	0.02%	0.29%	98.10%
	Total sRNAs	17685354	3204222	746684	7091	1823	593895	13131639
	Percent%	100%	18.12%	4.22%	0.04%	0.01%	3.36%	74.25%
PF4U	Unique sRNAs	5190025	19183	44498	2018	741	8246	5115339
	Percent%	100%	0.37%	0.86%	0.04%	0.01%	0.16%	98.56%
	Total sRNAs	15021455	2371366	455343	4551	1754	327426	11861015
	Percent%	100%	15.79%	3.03%	0.03%	0.01%	2.18%	78.96%
PF4S	Unique sRNAs	5998800	20816	65837	3572	1062	9985	5897528
	Percent%	100%	0.35%	1.10%	0.06%	0.02%	0.17%	98.31%
	Total sRNAs	18729181	2614487	913448	9899	2259	623069	14566019
	Percent%	100%	13.96%	4.88%	0.05%	0.01%	3.33%	77.77%

PF2U: diploid *P*. *fortunei* with0% NaCl; PF2S: diploid *P*. *fortunei* with 0.4% NaCl; PF4U: tetraploid *P*. *fortunei* with0% NaCl; PF4S: tetraploid *P*. *fortunei* with 0.4% NaCl.

### Identification of conserved miRNAs

The sRNAs that differed from known miRNAs by no more than two mismatches were defined as conserved miRNAs. We detected 53conserved miRNAs belonging to 17 miRNA families (12 of the miRNA families had not been found previously in *Paulownia*) (S3 Table). The abundances of the conserved miRNAs in the four libraries ranged from zero to 1,453,015, indicating that the expression of the miRNAs varied greatly. Members of the MIR166 family were the most abundant (approximately 64% on average), followed by MIR167-1 (approximately 18%) and MIR156 (approximately 15%). Except for the miRNAs with no family, members of the MIR4371family were the least with only six pfo-miR4371a/b sequences detected in the PF2Ulibrary. Among the miRNAs with no family, pfo-miR5239a/b were the most abundant with 74 all in four libraries, and pfo-miR5339 was the least with only five in the PF4Slibrary.Moreover, 24 miRNA*s were found among the conserved miRNAs, which confirmed the veracity of the conserved miRNAs. The numbers of miRNAs on the 5′and 3′armsof the stem-loop structures were 38 and 15respectively. Among the conserved miRNAs approximately 72% were 21-nt-long, followed by those that were 22- and 20-nt-long; only one conserved miRNA, pfo-miR169b, was 23-nt-long. Not all the conserved miRNAs were presented in all four libraries. For instance, pfo-miR3933a/b/c/d, pfo-miR1849, pfo-miR5339, and pfo-miR169b were expressed only in the PF4Slibrary and their expressions were low (not over 10); pfo-miR5021a/b and pfo-miR171a/b/c/d were expressed only in the PF2Slibrary with low expression. Moreover, pfo-miR399 was expressed only in untreated seedlings, while pfo-miR6269a/b and pfo-miR482e were expressed only in salt-treated seedlings. These results suggested that salt stress could influence plant growth and development by regulating their miRNAs. The expressions of miRNAs in the same family were different. For example, in the MIR156 family, pfo-miR156a/b/c were expressed similarly in all four libraries (341123 in the PF2U library, 268635 in thePF2S library, 406330 in thePF4U library, and 418990 in thePF4S library), while pfo-miR157was expressed differently (128016 in thePF2U library, 154352 in thePF2S library, 135041 in thePF4U library, and 189687 in thePF4S library).Additionally, in the MIR159 family, pfo-miR159a was expressed 2657 only in the PF4U library, while among the other members onlypfo-miR159b was not expressed in the PF4U library. Those findings implied that different family members may possess distinct functions.

### Identification of novel miRNAs

Among the remaining non-annotated sRNAs, 134 were predicted as potential novel miRNAs and 38 of them had corresponding miRNAs*, which validated our identification of novel miRNAs (S4 Table). The length the novel miRNAs ranged from 20 nt to 23nt; 21-nt-long novel miRNAs were the most abundant (approximately 49%), followed by 23-nt-long novel miRNAs (approximately 24%) and 22-nt-long novel miRNAs (approximately 17%).Among the novel miRNAs, 77 (approximately 57%)were on the 3′ arm of the stem-loop structure, the rest were on the 5′ arm. Moreover, the precursor stem-loop sequences were from 68-nt to 347-nt long with an average of 194nt.The minimum free energy (MFE)of their folded structures was from −19.8kcal/mol to −283.8kcal/mol with an average of−53.478kcal/mol. In addition, pfo-mir6a/b/c/d/e/f/g/h were the most abundant of the novel miRNAs, and their expressions were similar in each library (145381 in the PF2U library, 425766 in the PF2S library, 285551 in the PF4U library, and 273869 in the PF4S library) and averaged 282,641. The next most abundant novel miRNAs were pfo-mir3a/b (34576 in the PF2U library, 31947 in the PF2S library, 24266 in the PF4U library, and 29807 in the PF4S library), and the novel miRNAs with the lowest expressions (5) included pfo-mir51 and pfo-mir53 expressed in the PF2Slibrary, pfo-mir74, pfo-mir76, and pfo-mir89a/b/c expressed in thePF4Ulibrary, and pfo-mir88 and pfo-mir94 expressed in thePF4S library. Not all of the novel miRNAs were detected in all four libraries. Some were found in only one library, such as pfo-mir38a/b and pfo-mir90, which were expressed only in thePF2Sand PF4Slibraries, respectively. Some were found in only two libraries, such as pfo-mir68a/b, which were expressed only in thePF4U and PF4S libraries; and others were expressed in three of the four libraries, such as pfo-mir10, which was not detected only in thePF2S library, and pfo-mir12, which was not detected only in thePF4Ulibrary. Because of the range of their abundances and the different expression levels in the different libraries, we considered that the candidate novel miRNAs would have different functions in *P*. *fortunei* in response to salt stress.

### Differential expression analysis of miRNAs

All the conserved and novel miRNAs in the four libraries were normalized and their fold-changes and *P*-values were calculated. We compared the expression levels of the miRNAs in four comparisons:PF2S/PF2U, PF4U/PF2U, PF4S/PF4U, and PF4S/PF2S. MiRNAs with fold changes ≥1.0 or ≤−1.0, and *P*-values≤0.05 were considered to have significantly different expression levels. We found 23 conserved miRNAs belonging to 12 families that were significantly differentially expressed in the PF2S/PF2Ucomparison (S3 Table); 17 were upregulated and six were downregulated. Among them, pfo-miR399 had the lowest relative expression and pfo-miR6269a/b had the highest. We also found 31 upregulated novel miRNAs and 28 downregulated novel miRNAs in the PF2S/PF2Ucomparisonthat were significantly differentially expressed; pfo-mir42e/f had the lowest relative expression and pfo-mir3d/e had the highest (S4 Table). In the PF4S/PF4Ucomparison, we found 23 conserved miRNAs (13 upregulated and 10 downregulated) belonging to 12 families and 58 novel miRNAs (33 upregulated and 25 downregulated) that were significantly differentially expressed. In PF4U/PF2Ucomparison, we found 14 conserved miRNAs (three upregulated and 11 downregulated) belonging to 10 families and 78 novel miRNAs (42 upregulated and 36 downregulated) that were significantly differentially expressed. In PF4S/PF2S comparison, we found 28 conserved miRNAs (10 upregulated and 18 downregulated) belonging to 13 families and 74 novel miRNAs (45 upregulated and 29 downregulated) that were significantly differentially expressed.

Overall,10 conserved miRNAs from seven families and 10 novel miRNAs were found to be significantly differentially expressed in the PF2S/PF2U and PF4S/PF4Ucomparisons, suggesting that these miRNAs may be involved in the plants’ response to salt stress. Among these10 conserved and 10 novel miRNAs, six conserved miRNAs (pfo-miR159b, pfo-miR408a/b, pfo-miR477, pfo-miR482e, and pfo-miR530b) and seven novel miRNAs (pfo-mir15, pfo-mir28a/b, pfo-mir43a/b, pfo-mir56, and pfo-mir64) were also found to be significantly differentially expressed in the PF4S/PF2Scomparison. We removed the miRNAs that had the same trend of expression in the PF4S/PF2S and PF4U/PF2Ucomparisons to eliminate miRNAs that may be involved in ploidy differences. Finally, we identified three conserved miRNAs (pfo-miR408a/b and pfo-miR482e) and five novel miRNAs (pfo-mir28a/b, pfo-mir43a/b, and pfo-mir56) as the main salt-associated miRNAs that conferred higher tolerance in tetraploid *P*. *fortunei* than in diploid *P*. *fortunei*. In addition, we found that the numbers of novel miRNAs with significantly different expression levels among the four libraries were more than double those of the conserved miRNAs.

### Identification of miRNAs transcript targets by degradome analysis

**Fig 3:**
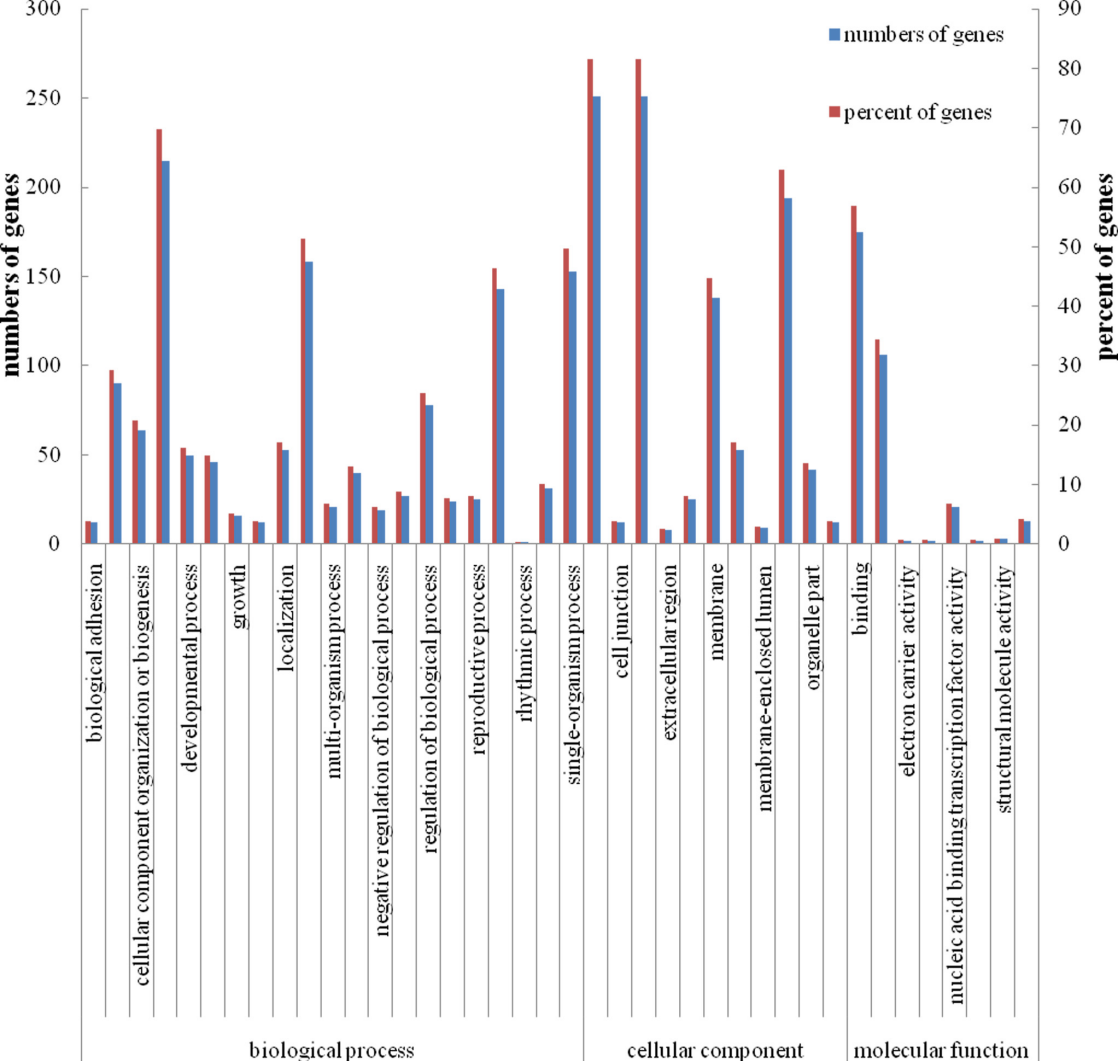
Gene Ontology analysis of miRNA targets in *P*. *fortunei*.

We used the degradome sequencing approach to identify targets of the identified *P*. *fortunei* miRNAs. PAIRFINDER predicted a total of 438 targets that could be cleaved by 12 of the conserved miRNA families and 15 of the novel miRNA candidates (S5 Table). The predicted target transcripts were pooled and grouped into three categories (I–III) based on their expression abundances; 169 (169 cleavage sites) were assigned to category I, 232(732 cleavage sites) to category II, and 74 (98 cleavage sites) to category III. Functional annotation was performed based on an analysis of assigned GO terms under the three GO categories, biological process, cellular component, and molecular function (Fig 3). In the GO analysis of the predicted target genes, the majority were annotated to be involved in cellular process, metabolic process, response to stimulus, single-organism process, cell, cell part, membrane, organelle, binding, and catalytic activity. The KEGG Pathway database was used to further classify the miRNA target genes. The KEGG Pathway annotations indicate that the target genes may be involved in a broad range of cell developmental processes, including energy metabolism, signal transduction, and transcriptional regulation, and suggest that they might have important functions during plant growth under salt stress. Among the miRNAs that were found to be significantly differentially expressed both in the PF2S/PF2U and PF4S/PF4Ucomparisons, two conserved miRNAs (pfo-miR159b and pfo-miR530b) and two novel miRNAs (pfo-mir28a/b) had predicted target genes. The annotations of these genes may provide insights in to the salt tolerance response in *P*. *fortunei*.

### Expression pattern analysis of *P*. *fortunei* miRNAs and their target genes

**Fig 4:**
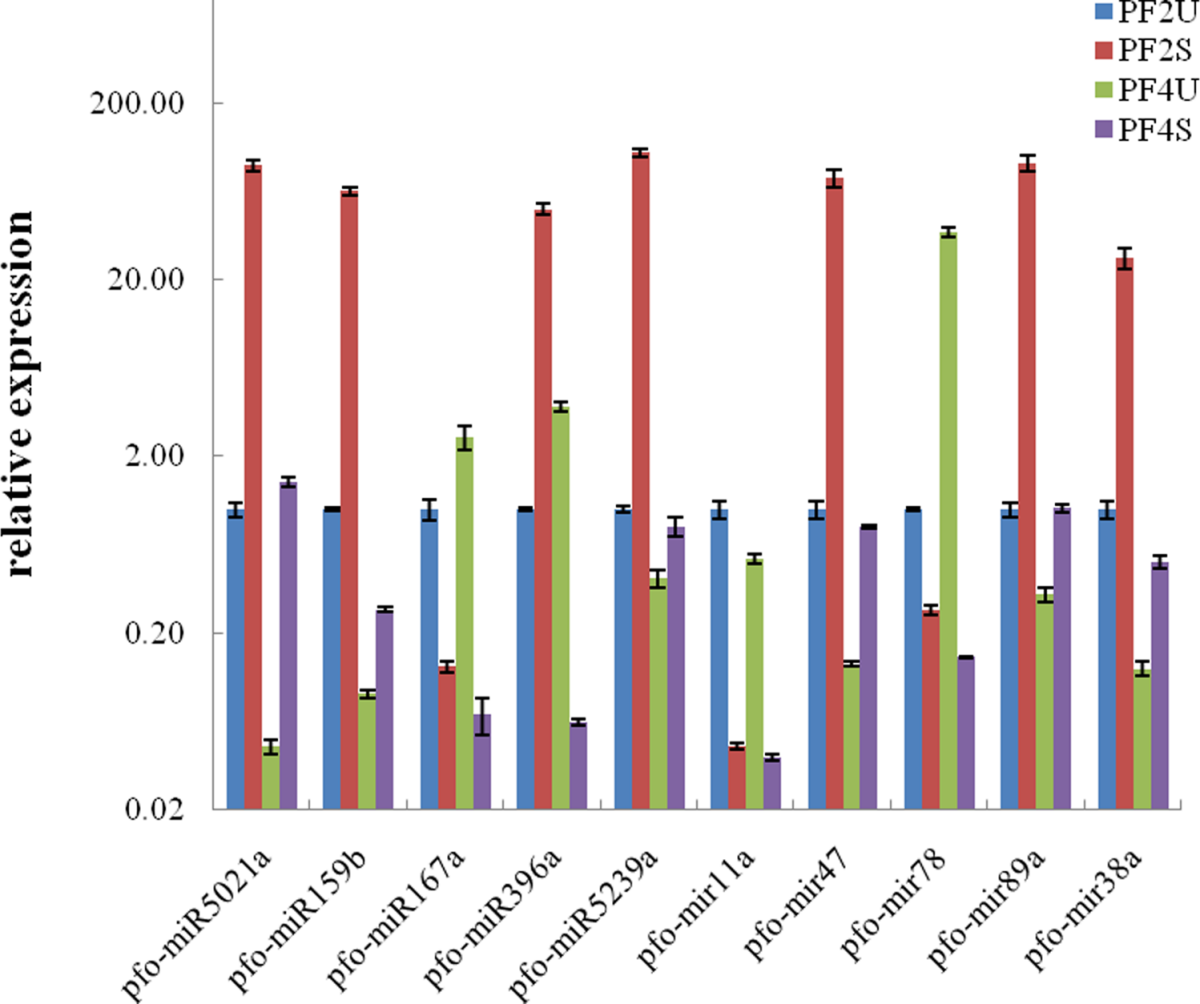
Relative expression levels of the miRNA in *P*. *fortunei* by qRT-PCR. PF2U: diploid *P*. *fortunei* with0% NaCl; PF2S: diploid *P*. *fortunei* with 0.4% NaCl; PF4U: tetraploid *P*. *fortunei* with0% NaCl; PF4S: tetraploid *P*. *fortunei* with 0.4% NaCl. The expression levels of miRNAs were normalized to U6. The normalized miRNA levels in the PF2U were arbitrarily set to 1.Standard error of the mean for three technical replicates is represented by the error bars.

To confirm the existence and expression levels of the miRNAs obtained from the high-throughput Illumina sequencing, 10 miRNAs with different expression levels with or without salt treatment were selected for qRT-PCR analysis (Fig 4). The expression patterns of the miRNAs acquired by the qRT-PCR show similar trends to their expression patterns in the four sequenced libraries. The expression levels of six of the miRNAs (pfo-miR5021a, pfo-miR159b, pfo-miR5239a, pfo-mir47, pfo-mir89a, and pfo-mir38a) were upregulated in the PF2S/PF2U and PF4S/PF4Ucomparisons, indicating that the expressions of these miRNAs may be increased by high salinity. The expressions of pfo-miR167a, pfo-mir11a, and pfo-mir78 were downregulated in the PF2S/PF2U and PF4S/PF4Ucomparisons, indicating that the expressions of these miRNAs may be decreased by high salinity. The different expression trends observed in the salt-treated plants suggest that the miRNAs could play different roles in *P*. *fortunei* growth and development. Further, we found that eight of the miRNAs, except pfo-miR167a and pfo-mir11a, were expressed most abundantly in the PF2S library, indicating that these miRNAs may play important roles in plant growth in salt-stressed diploid plants. Moreover, several miRNAs, such as pfo-miR5021a and pfo-mir11a that were not detected in thePF4Uand PF4Slibraries by Illumina sequencing were detected by qRT-PCR.

**Fig 5:**
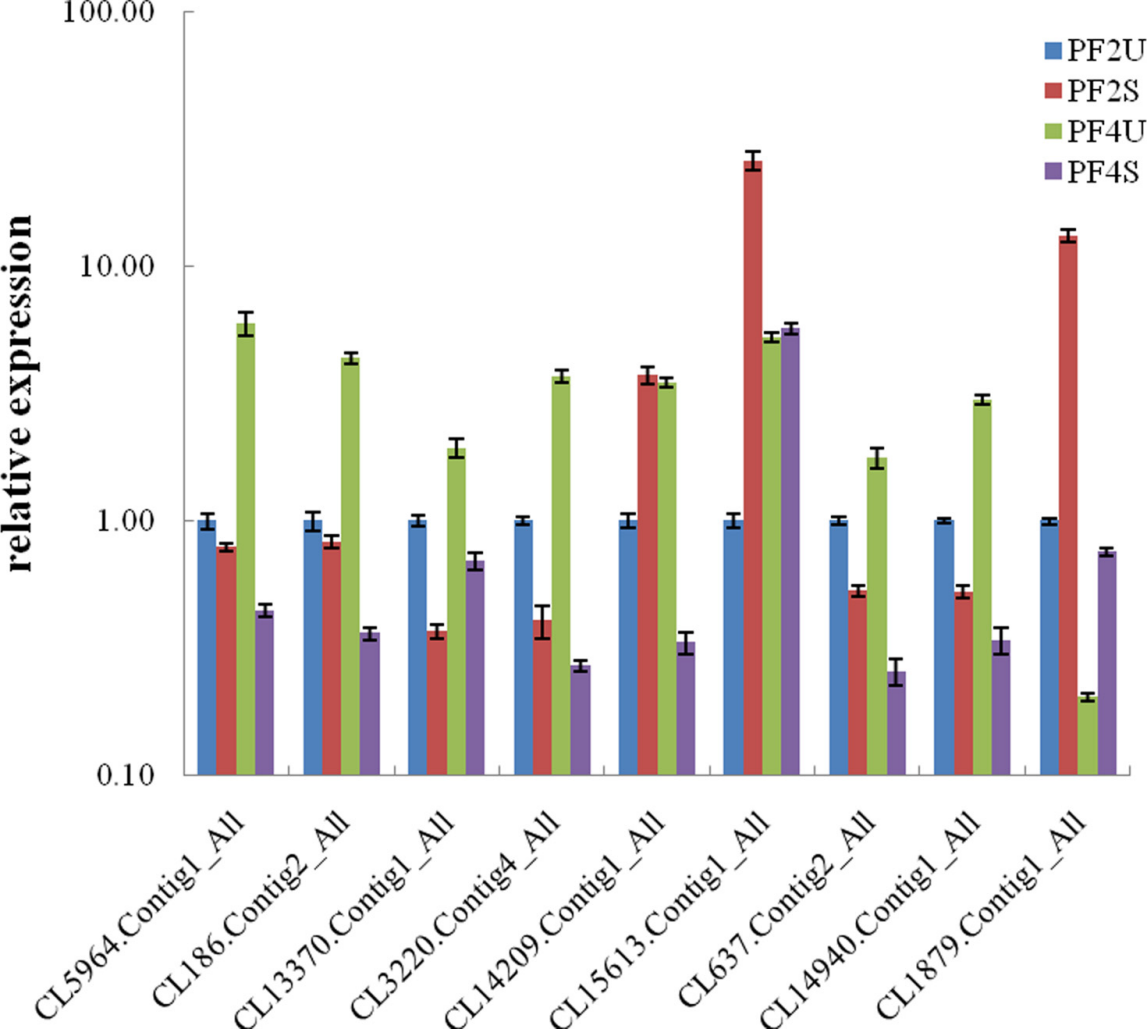
Relative expression levels of the target genes in *P*. *fortunei* by qRT-PCR. PF2U: diploid *P*. *fortunei* with0% NaCl; PF2S: diploid *P*. *fortunei* with 0.4% NaCl; PF4U: tetraploid *P*. *fortunei* with0% NaCl; PF4S: tetraploid *P*. *fortunei* with 0.4% NaCl. CL5964.Contig1_All targeted by pfo-miR159b. CL186.Contig2_All targeted by pfo-miR396a. CL13370.Contig1_All targeted by pfo-miR5021a. CL3220.Contig4_All targeted by pfo-miR5239a. CL14209.Contig1_All targeted by pfo-mir28a. CL15613.Contig1_All targeted by pfo-mir11a. CL637.Contig2_All targeted by pfo-mir47. CL14940.Contig1_All targeted by pfo-mir89a. CL1879.Contig1_All targeted by pfo-miR167a. Three independent biological replicates were performed. The expression levels of targets were normalized to 18SrRNA. The normalized miRNA levels in the PF2U were arbitrarily set to 1.Standard error of the mean for three technical replicates is represented by the error bars.

The expression patterns of nine of the predicted target genes were also verified by qRT-PCR (Fig 5). We found that the expression patterns of the target genes were inversely correlated with the expression patterns of the corresponding miRNAs. Five of the targets (CL5964.Contig1_All, CL13370.Contig1_All, CL3220.Contig4_All, CL637.Contig2_All, and CL14940.Contig1_All) were significantly more highly expressed in PF2S than PF2U and in PF4S than PF4U, while their corresponding miRNAs (pfo-miR159b, pfo-miR5021a, pfo-miR5239a, pfo-mir47, and pfo-mir89a, respectively) were upregulated in the PF2S/PF2U and PF4S/PF4Ucomparisons. Furthermore, CL15613.Contig1_All and CL1879.Contig1_All were both upregulated in the PF2S/PF2U and PF4S/PF4Ucomparisons, while the corresponding miRNAs, pfo-mir11a and pfo-miR167a, were downregulated in the PF2S/PF2U and PF4S/PF4Ucomparisons. The inverse correlation between the expression patterns of the miRNAs and their target genes indicated that these miRNAs negatively regulated their target genes.

## Discussion

Most plants can regulate their morphology, physiological, and biochemical pathways to adapt to high salinity. Our results showed that, with the salt treatment, the extracellular water potential was lower than in the cells, which led to cell water loss. The high ion content in the leaves of the salt-stressed plants caused the dissociation of chlorophyll and chloroplast proteins, after which the chlorophyll started to decompose[29, 30].To reduce salt stress, the majority of plants accumulate ions and organic material to reduce cell osmotic potential through osmotic regulation[31]. Under salt stress, enhanced membrane lipid peroxidation destroys membrane structure, which elevates plasma membrane relative permeability and MDA content. However, increased antioxidant activity of related enzymes could remove excess oxygen free radicals, hydrogen peroxide, and hydroxyl free radical from the cells[32, 33].

In our study, the eight physiological indices that we measured were all lower in tetraploid than in diploid *P*. *fortunei*, which showed that tetraploid had more significant growth than diploid in the salt-treated and untreated samples. Tetraploid seedlings exhibited higher relative water content, chlorophyll content, soluble sugar content, proline content, SOD activity, and soluble protein content, and lower MAD content and relative electrical conductivity than diploid seedlings in response to salt stress. These physiological parameters implied that tetraploid might be better capable of surviving salty conditions, which is consistent with the findings of previous studies[11, 34, 35].

To understand the salt tolerance of diploid and tetraploid *P*. *fortunei* more deeply at the molecular level, we performed Illumina sequencing and degradome analysis. Ten conserved and ten novel miRNAs that were significantly differentially expressed in both the PF2S/PF2U and PF4S/PF4Ucomparisons were predicted to be involved in the high-salinity response. Among these 20 miRNAs, we selected six conserved miRNAs (pfo-miR159b, pfo-miR408a/b, pfo-miR477, pfo-miR482e, and pfo-miR530b) and seven novel miRNAs (pfo-mir15, pfo-mir28a/b, pfo-mir43a/b, pfo-mir56, and pfo-mir64) that were also significantly differentially expressed in the PF4S/PF2Scomparison. Predicted target genes of pfo-miR159b, pfo-miR530b, and pfo-mir28a/b were identified for further study by degradome sequencing. To select the main salt-associated miRNAs that conferred higher tolerance in tetraploid *P*. *fortunei* than in diploid *P*. *fortunei*, we identified three conserved miRNAs (pfo-miR408a/b and pfo-miR482e) and five novel miRNAs (pfo-mir28a/b, pfo-mir43a/b, and pfo-mir56) that had the opposite trend of expression in the PF4U/PF2Uand PF4S/PF2S comparisons.

The pfo-miR159b miRNA, which was upregulated in the PF2S/PF2U and PF4S/PF4Ucomparisonsand downregulated in the PF4U/PF2U and PF4S/PF2Scomparisons, targeted genes encoding a GAMYB-like transcription factor. GAMYB is the activator of gibberellin (GA)-regulated genes. GAs play important roles in plant growth and development including floral initiation, stem elongation, anther development, and seed development[36].Comparing tetraploid with diploid, we found that the expression trends of GAMYB-like transcription factor were consistent in different *Paulownia* species at same stages and inconsistent in same *Paulownia* species at different stages [14, 17].Ren et al.[37]reported that miR319 targeted genes encoding MYB-like DNA-binding domain proteins, indicating thatmiR159was not the only miRNA that could target genes encoding GAMYB-like transcription factors. Yang et al. showed that increased expression of members of the stu-miR159 family in potato decreased the expression of the GAMYB-like transcription factor family genes in response to drought stress [38]. The different expression levels of miR159 between our study and that of Yang et al. may indicate that the regulation of GAMYB-like genes by miR159in salt- or drought-stressed plants was complex. The stress hormone abscisic acid (ABA)also plays a vital role in plants under abiotic stress [2, 39]. In *P*. *fortunei*, GA and ABA may regulate the GAMYB-like transcription factor and influence its growth and development in response to salt stress. Under highly salinity, the expressions of GAMYB-like genes predicted to be targeted by pfo-miR159b increased; however, the increased expression was lower in tetraploid than in diploid, indicating that tetraploid could respond better to salt stress than diploid.

Apart from the GAMYB-like genes, genes encoding WRKY and basic leucine zipper (bZIP) transcription factors also were detected by the degradome sequencing. These genes were predicted to be targeted by pfo-mir73. WRKY transcription factors modulate many processes in plants [40], and have been shown to be the activators or repressors of ABA signaling [39]. For example, *GsWRKY20* was shown to enhance the drought tolerance and increase the negative regulators or decrease the positive regulators of ABA signaling[41]. Whether WRKY acts as an activator or repressor, its increased or decreased expression could impact the ABA response to salt stress. Golldack et al. found that WRKY proteins may take part in plant growth and development under salt and drought environments[42–44]. WenBo et al.[45]found that AtWRKY2 was influenced by salt treatment in *Arabidopsis* plants and Zhao et al. [46]indicated that PsnWRKYs were downregulated in *Populus simonii*×*Populus nigra*. These results are in accord with our findings that pfo-mir73 targeted genes encoding WRKY, and was downregulated in the PF4S/PF4Ucomparison and upregulated in the PF4U/PF2U comparison. Thus, we predicts that WRKY, which was encoded by a gene targeted by pfo-mir73, was the repressor of ABA in *P*. *fortunei*; that is, ABAR (Mg-chelatase H subunit/putative ABA receptor) bound ABA by spanning the chloroplast envelope and the cytosolic C-terminus, and also interacted with WRKY. After the salt treatment, the elevated membrane relative permeability of the plasma membrane would make it easier for ABAR and ABA to span the chloroplast envelope. The high levels of ABA added WRKY from the nucleus to the cytosol to interact with ABAR, so the repression of ABA-responsive genes in the nucleus was reduced. Thus, the activator of ABA-responsive genes GAMYB, which was targeted by pfo-miR159b, was increased in *P*. *fortunei* under salt stress[39].Furthermore, the ZIP transcription factor family is abundant and diverse in plants [47], such as *Arabidopsis* [48], rice [47, 49], *Sorghum*[50], and maize [51, 52]. BZIPs are known to be involved in plant growth and development, and in adversity stress responses; for instance, bZIPs have been associated with energy metabolism[53], floral induction[54–57], photomorphogenesis [58–60], high-salinity stress [49, 61, 62], drought stress [63, 64], temperature stress [65, 66], pathogen infection[67, 68], ABA signaling [63],and light signaling [69]. Jiet al.[70]demonstrated that ABA and salt stress were not positively or negatively regulated together during plant growth and development. Thus, the roles of bZIPs in *P*. *fortunei* are likely to be complex and dynamic in response to salt stress, and the causes need to be investigated further.

The expression levels of the conserved miRNA pfo-miR530b were upregulated in the PF2S/PF2U and PF4S/PF2S comparisons and downregulated in the PF4S/PF4U comparison. However, although the expression of pfo-miR530b decreased in tetraploid and increased in diploid under salt treatment, its expression was still higher in tetraploid than diploid. The three predicted target genes of pfo-miR530b did not seem to encode any known genes; therefore, we speculated that the target genes of pfo-miR530b may encode a novel protein related to resistance to salinity.

Pfo-mir28a/b were identified as novel miRNAs that were downregulated in the PF2S/PF2Ucomparison and upregulated in the PF4S/PF4U and PF4S/PF2Scomparisons. These miRNAs had two predicted target genes that could encode a disease resistance(R) protein and an ATPase. In salt-treated diploid and tetraploid plants, pfo-mir28a/b showed differential expression and the expressions of the disease resistance protein and ATPase showed a greater increase in tetraploid compared with diploid. This finding is consistent with the results that MDA, soluble sugar, and proline contents increased in response to salt stress and this effect was more enhanced in tetraploid than in diploid. Thus, we conjectured that the disease resistance protein and ATPase may be related to the salt stress response in *P*. *fortunei*, again indicating tetraploid had better salt resistance than diploid. The most numerous disease resistance proteins class was represented by genes that code for proteins containing a nucleotide-binding site (NBS) and leucine-reach repeats (LRRs)[71–73]. NBSs are found in diverse proteins and are required for ATP and GTP binding[74, 75],indicating that disease resistance proteins may be associated with ATPases. LRRs are highly adaptable structural domains involved in protein-protein interactions that can evolve very different binding specificities [76]. Marone [76] showed that NBS-LRR genes can enhance the resistance of plants to disease-causing pathogens. Membrane-bound ion channel ATPases import many of the metabolites necessary for cell metabolism and export toxins, wastes, and solutes that can hinder cellular processes. The upregulation ofpfo-miR530bmay have added more ATPases that could import more metabolites to reduce cell osmotic potential for water loss reduction. This finding is consistent with the physiological indices measured in our study. In *P*. *fortunei*, ATPases may hinder biotic or abiotic stress through R genes and undergo complex interactions to resist salt stress.

The predicted targets of pfo-mir73 and pfo-miR482aencode NBS-LRR class disease resistance proteins. Their expressions were opposite (pfo-mir73 was upregulated in PF4U/PF2U and downregulated in PF4S/PF2S, pfo-miR482a was only downregulated in PF4U/PF2U) and different from the expressions of pfo-mir28a/b, which further confirmed the disease resistance protein and ATPase were involved in complex dynamic regulation of the response of *P*. *fortunei* to salt resistance. Furthermore, the expression of pau-miR482bwhich targeted disease resistance proteins in tetraploid *P*. *tomentosa* was lower than in diploid *P*. *tomentosa*[13]; the expressions of pas-miR482a/b/c-3pin tetraploid *P*. *australis* were all higher than in diploid *P*. *australis*[17]. The different expression in various *Paulownia* species showed thatmiR482was ubiquitous and may play a complex role in plants.

In our study, the predicted targets of pfo-miR167a/b and pfo-mir73 encode auxin response factors (ARFs). ARFs act as regulators in plant growth and development processes and in responses to environmental stress conditions [77–79]. Auxin is involved in signaling cell division, elongation, and differentiation at the cellular level, and plays an important role in lateral root formation, apical dominance, and tropisms [80]. The Aux/IAA, GH3, and SAUR gene families are regulated by auxin[81].Different results have been reported for different plants under salt stress. In previous studies, the expression of pto-miR160g was downregulated in *P*. *tomentosa*[37]; vun-miR160a was reported to be upregulated in *Vigna*[82]; and miR160a and miR160b were induced by 5hours of salt treatment but reduced by 24hours of salt treatment in maize. In this study, the expressions of pfo-miR167a/b were downregulated only in the PF4S/PF4U comparison, while pfo-mir73 was downregulated in the PF4S/PF4Ucomparison and upregulated in the PF4U/PF2U comparison. At different stages, the expression trends of pas-miR167 were inconsistent in tetraploid *P*. *australis* compared with diploid *P*. *australis*[17]. Together, these findings showed that ARF expression was decreased in tetraploid under high salinity, which indicated that ARF may regulate various auxins through different pathways in response to highly salinity.

We also found that pfo-mir73 targeted genes encoding F-box, assembly factor, and proline. F-box is one of three components of the SCF complex, which mediates ubiquitination of proteins targeted for degradation by proteasomes. F-box proteins also are involved in cellular functions such as signal transduction and regulation of the cell cycle[83]. In plants, many F-box proteins are represented in gene networks broadly regulated by miRNA-mediated gene silencing via RNA interference [84]. In response to salt stress, F-box proteins have been found to be important and their expressions were reported to be different in different plants [85]. In *P*. *tomentosa*, F-box protein encoding genes were significantly downregulated in response to salt stress [37], while in salt-stressed *Arabidopsis*, F-box proteins were found to be induced [86, 87].Thus, it is likely that F-box proteins play different roles in different plants. Regarding the assembly factor, Cheng[88] identified nine differentially expressed proteins involved in photosynthesis, redox homeostasis, stress/defense, carbohydrate and energy metabolism, protein metabolism, signal transduction, and membrane transport. One of these proteins was the photosystem II stability/assembly factor HCF136, which was found to decrease in response to salinity stress. HCF136 has been shown to play a role in the light reactions and in the Calvin cycle[89]. Primary metabolites are necessary for growth, development, and response to the environment [90]. In tetraploid Ziyang xiangcheng [91], stress-related metabolites such as sucrose, proline, and GABA were found to be upregulated, which is similar to the results of our study where the higher the salinity, the higher the soluble sugar and proline contents. In contrast, miRNA pfo-mir73, which targeted genes encoding proline, was downregulated in PF4S/PF4U. We considered that the pfo-mir73expression levels that we obtained were not accurate, because they indicated that the soluble sugar and proline contents were higher in tetraploid than in diploid; the finding that the pfo-mir73 target genes, which encode proline synthesis, were upregulated in PF4U/PF2Ualso confirmed our assumption. Although pfo-mir73 was predicted to play various roles in response to salt stress, its expression was very low probably because of faults in the miRNA extraction process or in the degradome sequencing.

In summary, we measured physiological indices, identified conserved and novel miRNAs by high-throughput sequencing, and analyzed their expression levels in diploid and tetraploid salt-stressed *P*. *fortunei* seedlings. GO and KEGG pathway analyses were performed to predict the functions of the miRNA target genes. We identified miRNAs that were associated with higher salt tolerance in tetraploid *P*. *fortunei* than in diploid *P*. *fortunei* and analyzed miRNAs at physiological and molecular levels. Our results will contribute to further research into the response mechanisms of *P*. *fortunei* to salt stress.

## Supporting Information

S1 Table Primers of miRNAs used for qRT-PCR analysis. (XLSX)

S2 Table Primers of target genes used for qRT-PCR analysis. (XLSX)

S3 Table Conserved miRNAs identified from *P*. *fortunei*. PF2U: diploid *P*. *fortunei* with0% NaCl; PF2S: diploid *P*. *fortunei* with 0.4% NaCl; PF4U: tetraploid *P*. *fortunei* with0% NaCl; PF4S: tetraploid *P*. *fortunei* with 0.4% NaCl.
(XLSX)

S4 Table Novel miRNAs identified from *P*. *fortunei*. PF2U: diploid *P*. *fortunei* with0% NaCl; PF2S: diploid *P*. *fortunei* with 0.4% NaCl; PF4U: tetraploid *P*. *fortunei* with0% NaCl; PF4S: tetraploid *P*. *fortunei* with 0.4% NaCl.
(XLSX)

S5 Table Identified targets of miRNAs involved in *P*. *fortunei* by degradome analysis. (XLSX)
